# The role of neuroinflammation in the transition of acute to chronic pain and the opioid-induced hyperalgesia and tolerance

**DOI:** 10.3389/fphar.2023.1297931

**Published:** 2023-12-15

**Authors:** Marco Echeverria-Villalobos, Victor Tortorici, Beatriz E. Brito, David Ryskamp, Alberto Uribe, Tristan Weaver

**Affiliations:** ^1^ Anesthesiology Department, The Ohio State University Wexner Medical Center, Columbus, OH, United States; ^2^ Neuroscience Laboratory, Faculty of Science, Department of Behavioral Sciences, Universidad Metropolitana, Caracas, Venezuela; ^3^ Neurophysiology Laboratory, Center of Biophysics and Biochemistry, Venezuelan Institute for Scientific Research (IVIC), Caracas, Venezuela; ^4^ Immunopathology Laboratory, Center of Experimental Medicine, Venezuelan Institute for Scientific Research (IVIC), Caracas, Venezuela; ^5^ College of Medicine, The Ohio State University, Columbus, OH, United States

**Keywords:** acute pain, chronic pain, neuroinflammation, dysbiosis, gliosis, inflammatory mediators, opioids

## Abstract

Current evidence suggests that activation of glial and immune cells leads to increased production of proinflammatory mediators, creating a neuroinflammatory state. Neuroinflammation has been proven to be a fundamental mechanism in the genesis of acute pain and its transition to neuropathic and chronic pain. A noxious event that stimulates peripheral afferent nerve fibers may also activate pronociceptive receptors situated at the dorsal root ganglion and dorsal horn of the spinal cord, as well as peripheral glial cells, setting off the so-called peripheral sensitization and spreading neuroinflammation to the brain. Once activated, microglia produce cytokines, chemokines, and neuropeptides that can increase the sensitivity and firing properties of second-order neurons, upregulating the signaling of nociceptive information to the cerebral cortex. This process, known as central sensitization, is crucial for chronification of acute pain. Immune-neuronal interactions are also implicated in the lesser-known complex regulatory relationship between pain and opioids. Current evidence suggests that activated immune and glial cells can alter neuronal function, induce, and maintain pathological pain, and disrupt the analgesic effects of opioid drugs by contributing to the development of tolerance and dependence, even causing paradoxical hyperalgesia. Such alterations may occur when the neuronal environment is impacted by trauma, inflammation, and immune-derived molecules, or when opioids induce proinflammatory glial activation. Hence, understanding these intricate interactions may help in managing pain signaling and opioid efficacy beyond the classical pharmacological approach.

## 1 Introduction

According to the International Association for the Study of Pain (IASP), chronic pain persists or recurs for over 3 months and is a leading source of human suffering and disability (Treede et al., 2019). A cornucopia of etiological factors has been implicated in the genesis of chronic pain. It may be the primary symptom of a non-specific underlying disease (i.e., fibromyalgia), a sign of a progressive local/systemic disorder (i.e., rheumatic inflammatory diseases, Diabetes Mellitus), or secondary to direct nerve injury (Vergne-Salle and Bertin, 2021). More recently, a new pain mechanism identified as “nociplastic pain” has been proposed. It occurs in individuals with an abnormally elevated nociception sufficient to activate peripheral nociceptors without clear evidence of tissue damage, neural injury, or disease (Kosek et al., 2016). Although central sensitization is not included in the definition of nociplastic pain, patients with this condition frequently refer to signs of central sensitization, suggesting that nociceptive and neuroplastic pain may coexist (Kosek et al., 2016). Unlike acute pain, which functions as a defensive mechanism, chronic pain is considered maladaptive because it does not provide additional protective or recuperative benefits (Walters, 2019). Peripheral sensitization is the most common mechanism of pain initiation; nonetheless, the chronicity of pain is mainly determined by central sensitization. The neuroinflammatory response also gives rise to structural and functional maladaptive changes in the peripheral and central somatosensory pathways, modifying the pain-signaling process. The existing evidence suggests that activation of glial cells and other non-neuronal cells, such as immune cells (neutrophils, macrophages, T-cells, and mast cells), leads to increased production and release of proinflammatory mediators that play a crucial role in developing and maintaining neuropathic and chronic pain (Hains et al., 2006; Chiu et al., 2012; Du et al., 2023).

The main goal of this article is to review the current evidence on the molecular mechanisms that support the role of neuroinflammation as an important etiopathogenic factor in the transition from acute postoperative pain to chronic postoperative pain (CPSP). Similarly, the neuroimmune interactions associated with the decrease in the analgesic effect of opioid drugs are considered, including those involved in the development of opioid-induced inflammation, opioid tolerance, and paradoxical hyperalgesia.

## 2 The leading role of the immune response and glial activation in the transition from acute to neuropathic and chronic pain

Acute pain after surgery is almost a sine *qua non* condition; however, this kind of pain is ineffectively treated. It transitions to a state of chronic pain, often with neuropathic characteristics, that is refractory to treatment with opioids (Haroutiunian et al., 2013; Honkanen et al., 2021). The incidence of pain persisting beyond the period of wound healing after surgery is approximately 10%–85%, depending on the type of surgery. This has been typified as CPSP, with 5%–10% of cases rating the pain as severe, affecting patients quality of life and creating a significant economic burden for the health systems (Kehlet et al., 2006; Johansen et al., 2012; Jin et al., 2021; Jin et al., 2023). The most recent revision of the International Classification of Diseases (ICD-11) defines CPSP as “pain developing or increasing in intensity after a surgical procedure, in the area of the surgery, persisting beyond the healing process (i.e., at least 3 months) and not better explained by another cause such as infection, malignancy or a pre-existing pain condition”. Similarly, the IASP defines it as “chronic pain that develops or increases in intensity after a surgical procedure or a tissue injury and persists beyond the healing process, i.e., at least 3 months after the surgery or tissue trauma” (Schug et al., 2019).

**FIGURE 1:**
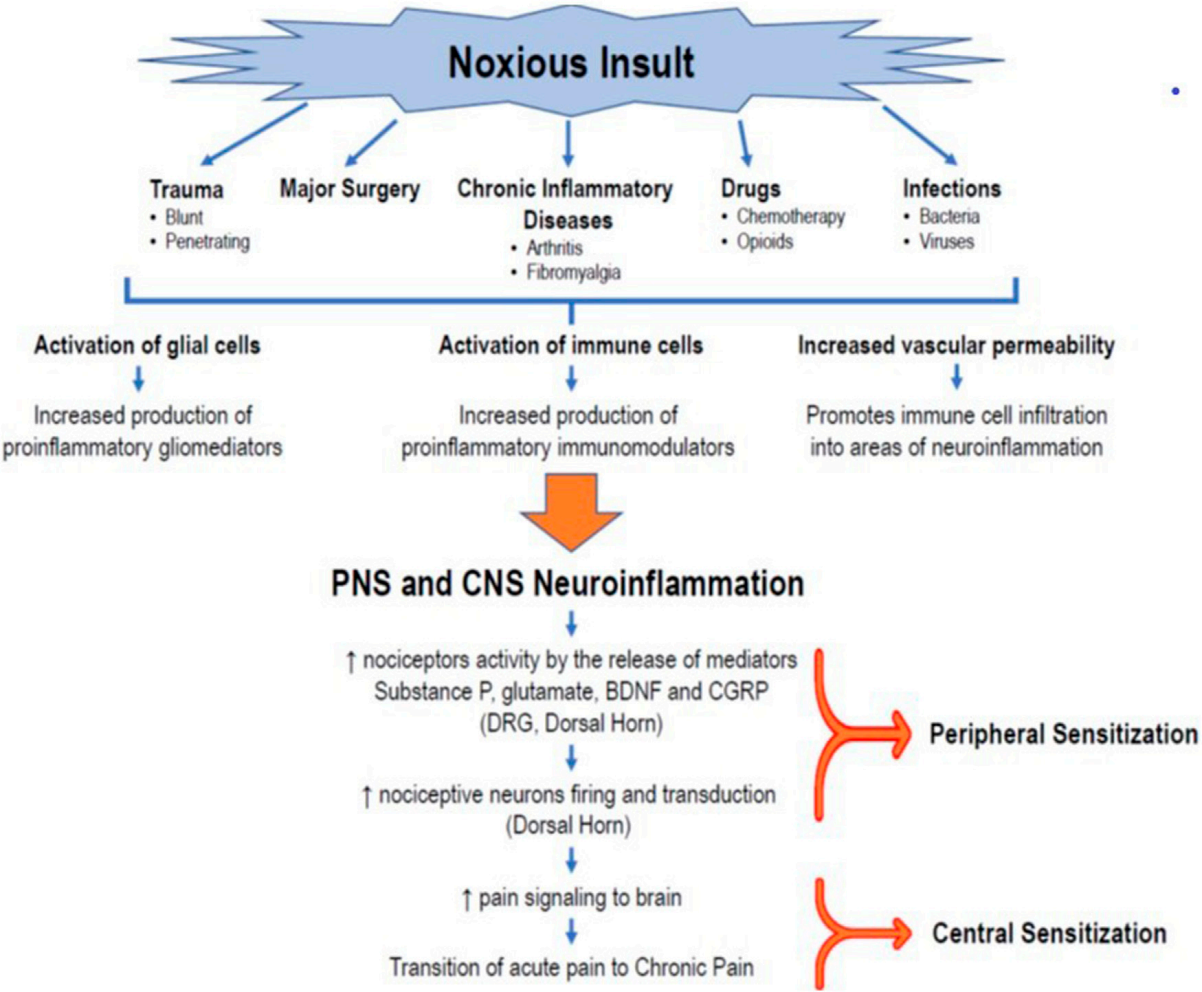
Role of neuroinflammation in the transition to CPSP. The occurrence of a noxious insult, such as trauma, major surgery, chronic inflammatory diseases, drugs (chemotherapy, opioids), or infections, triggers an initial immunoinflammatory response that, in turn, promotes the activation of glial cells (microglia, astrocytes, oligodendrocytes) in the peripheral sensory system, creating a state of peripheral neuroinflammation. At the same time, the production and release of inflammatory mediators produce changes in vascular permeability that facilitate the infiltration of immune cells that activate the glial cells of the CNS, generating the release of more proinflammatory mediators and a process of neuroinflammation in the CNS. This milieu of cytokines, chemokines, neuropeptides (substance P, CGRP), and neurotrophic factors leads to changes in the plasticity of second-order neurons in the posterior horn of the spinal cord. This results in an exaggerated firing activity and amplification of nociceptive signaling in the DRG and the posterior horn of the spinal cord, establishing the peripheral sensitization phenomenon. The transmission of painful signals to the brain, together with the process of neuroinflammation, keeps the nociceptor neurons of the brain’s pain centers stimulated, giving rise to a state of central desensitization, and promoting the transition from acute pain to neuropathic or chronic pain. **BDNF** = brain-derived neurotrophic factor; **CGRP =** calcitonin gene-related peptide; **CNS =** central nervous system, **DRG =** dorsal root ganglion.

Currently, overwhelming evidence supports the fundamental role of neuroinflammation in pain; however, its etiopathogenic mechanisms are not yet fully elucidated. A neuroinflammatory state is characterized by the activation of glial cells, generation of proinflammatory mediators (cytokines and chemokines, among others), and changes in the vasculature, resulting in increased permeability and leukocytic infiltration, as well as alterations in gene expression (Ji et al., 2006; Ji et al., 2018; Donnelly et al., 2020). Increasing evidence shows that glial tissue activation (including microglia, astrocytes, oligodendrocytes, satellite glial cells, and ependymal cells) plays a pivotal role in developing peripheral and central sensitization (Figure 1).

### 2.1 Role of immune cells

In the perioperative period, harmful events, such as surgical incisions and organ manipulation, cause tissue and nerve damage, which triggers the activation of immune cells, glial cells, and nociceptive neurons. These cells then set forth the release of pro-inflammatory mediators causing a pervasive state of inflammation in the peripheral and central nervous system. If the inflammation is not adequately resolved and central sensitization is established, the nociceptive state may progress from acute postoperative pain to CPSP once (Ji et al., 2006; Ji et al., 2013; Ji et al., 2016; Pinho-Ribeiro et al., 2017; Ji et al., 2018; Ji, et al., 2019). Additionally, recent evidence from preclinical studies shows that epidermic resident cells, keratocytes, and dendritic cells (DCs) play an active role in inflammatory pain arising from surgical tissue injury as well as in the development of neuropathic pain and CPSP (Manjavachi et al., 2014; Hesselink et al., 2016; Guo et al., 2020; Silva et al., 2022). Keratocytes lie close to sensory afferent nerves and when stimulated by surgical incision initiate a nociceptive response mediated by the production and release of various neuroactivator agents such as cytokines (TNF-α and IL-1β), calcitonin gene-related peptide receptor (CGRP), acetylcholine, adenosine triphosphate (ATP), nerve growth factors, neuropeptides, and other neurotransmitters contributing to peripheral neuroinflammation (Guo et al., 2020). Studies in animal models have shown that activated keratocytes can also elicit afferent firing and sensitization, resulting in postsurgical incisional hypersensitivity (Hesselink et al., 2016; Ritter-Jones et al., 2016; Guo et al., 2020; Silva et al., 2022). DCs and Langerhans cells are another group of skin-resident cells that become upregulated after tissue injury and can directly activate peripheral nociceptive neurons (nociceptors), releasing chemokines CCL17 and CCL22 through the shared receptor CCR4, contributing to neuroinflammation and postoperative pain (Silva et al., 2022).

Activated immune cells (macrophages, neutrophils, T-cells, and mast cells) also produce and release pro-inflammatory mediators that interact with peripheral nerve terminals and their somas located in the dorsal root ganglia (DRG), which relay in laminae I and II of the dorsal horn of the spinal cord (Pinho-Ribeiro et al., 2017). DRG macrophages are critical contributors to the initiation and persistence of neuropathic pain in male and female mice, without the sexual dimorphism reported in microglial cells (Yu et al., 2020). It has been recently demonstrated that immediately after a nerve injury, there is a significant proliferation of resident macrophages in the DRG ipsilateral to the nerve lesion, which produces proinflammatory cytokines such as TNF-α, IL-1β, and HMGB1 (Hu and McLachlan, 2003; Guimarães et al., 2023). The proximity of macrophages to the cell bodies of primary nociceptors in the DRG facilitates the activation of primary sensory neurons by the proinflammatory cytokines (Chen et al., 2018)”.

Macrophages are tissue immune cells that are stimulated early when nerve injury occurs. Until recently, macrophages were thought to be two polarized forms of immune cells (M1 and M2) with distinct phenotypic qualities and functional properties. Accordingly, M1 activity inhibited cell proliferation and caused tissue damage, whereas M2 activity promoted cell proliferation and tissue repair. However, recent evidence has shown that the expression of specific polarization markers for M1/M2 phenotypes *in vitro* observations can be influenced not only by a specific stimulus but even by a specific stimulation time sequence (Purcu Duygu et al., 2022).

*In vivo*, the situation is even more complex, as there is a wide range of different macrophages depending on the conditions of the microenvironment (Strizova et al., 2023). Moreover, most surface markers identified on macrophages generated *in vitro* do not translate to the situation *in vivo* (Orecchioni et al., 2020). These facts suggest that macrophages exhibit phenotypic plasticity and can adopt different activation states in response to different conditions. Therefore, the current phenotypic status encompasses an expanded spectrum of possibilities that includes multiple subsets of macrophages identified by their different activation states, functional properties, and surface marker expression. To further complicate matters, certain subsets of macrophages may not be completely distinct from one another and may share common functional activities.

These dualistic implications also apply to microglia, the macrophages of the brain, whose phenotype is also influenced by the central nervous system microenvironment. The repertoire of microglial states and functions goes beyond the dichotomy of “resting vs activated,” “M1 vs M2,” or “good vs bad microglia,” as demonstrated by the impressive work of Paolicelli et al. (2022), a group of multidisciplinary experts who advance the understanding of microglial states as a dynamic concept, emphasize the importance of considering microglial function, and provide a new conceptual framework for this type of dichotomy.

Therefore, the large phenotypic heterogeneity exhibited by macrophages might lead to oversimplification in light of recent observations. In the transitional phase, it might be appropriate, albeit risky, to describe macrophages as M1-like and M2-like to define their different roles rather than using the conventional M1 and M2 nomenclature (Strizova et al., 2023). However, the latter would be very helpful when referring to previous work reporting on the dichotomy.

It is normally accepted that M1-like macrophages liberate proinflammatory cytokines (TNFα, IL-1β, IL-6), chemokines (CCL2, CCL3, CCL4), and nitric oxide (NO). They also respond to damage-associated molecular patterns (DAMPs), and pathogen-associated molecular patterns (PAMPs) such as lipopolysaccharide (LPS). This results in further recruitment and activation of other immune cells (neutrophils, monocytes, mast cells, T-cells) and unmyelinated afferents, causing the sensitization of primary nociceptive neurons at the DRG (Nicol et al., 1997; Oh et al., 2001; Obreja et al., 2002; Zhang and An, 2007). In a rodent model of arthritis, a proinflammatory macrophage phenotype similar to the M1-like subtype was found in the rat DRG, and activation of these macrophages stimulated DRG neurons to release calcitonin gene-related peptide (CGRP), which plays an active role in the persistence of the pain state (Massier et al., 2015). Conversely, M2-like macrophages promote analgesia in response to IL-4, which stimulates the production of high levels of anti-inflammatory cytokines, such as IL-10 (Celik et al., 2020). IL-4 has also been shown to induce the M2-like subtype to produce endogenous opioid peptides that bind to peripheral opioid receptors, further contributing to pain relief by deactivating neuropathy-triggered mechanical hypersensitivity (Pannell et al., 2016; Celik et al., 2020). Furthermore, the action of the cytokine IL-4 also promotes the transcription of M2-like associated genes while reducing the transcription of M1-like associated genes (Kiguchi et al., 2015).

Neutrophils are polymorphonuclear leukocytes representing the first-line cells in the innate immune response to tissue damage or infections. However, their role in neuroimmune interactions has only recently been studied in greater detail (Chavan et al., 2017; Pavlov et al., 2018). Neutrophils are almost always absent around the intact nerves. However, when tissue damage occurs, they become activated by locally produced mediators such as proinflammatory cytokines, growth factors, leukotrienes B4 (LB4), DAMPs, and prostanoids such as prostaglandin E2 (PGE2). That milieu of mediators enhances neutrophil migration toward the sites of inflammation. Subsequent neutrophil infiltration of afferent nerve endings activates and sensitizes the terminals of the peripheral nociceptors and increases vascular permeability (Kalaczkowska and Kubes, 2013). Once activated, neutrophils continue releasing mediators, contributing to nociceptive amplification (Levine et al., 1984; Grace et al., 2014). Several studies have demonstrated that endoneural neutrophil invasion of peripheral nerves and DRGs occurs after induced chronic constriction injury of peripheral nerves. This neutrophil infiltration is correlated with an increase in the production of monocyte chemoattractant protein-1 (MCP-1, also known as CCL2) and neutrophil-derived elastase. Both are regarded as essential mediators in the development of hyperalgesia in animal models of neuropathic pain (Perkins and Tracey, 2000; Morin et al., 2007; Bali and Kuner, 2017). Contrarily, some researchers have questioned the role of cytokines in mechanical hyperalgesia while assigning a more important role to the release of bradykinin, PGE2, and sympathomimetic amines such as adrenaline, epinephrine, and norepinephrine (Green, 1974; Cunha et al., 2008). Neutrophils also participate in the process of neuroinflammation and post-incisional hypersensitivity through the production of other mediators such as metalloproteases, reactive oxygen species (ROS), hydrogen, and endothelins, which further increase nociceptors excitability (Steen et al., 1992; Wang et al., 2004; Woo et al., 2004; Mujenda et al., 2007). In the CNS, neutrophil infiltration occurs as a result of changes in the permeability of the blood-brain barrier (BBB) secondary to the neuroinflammation process, and the chemoattractant effect of chemokines CXCL2 and CXCL6, released by meningeal mast cells and astrocytes. This neutrophilic invasion causes demyelination and axonal damage (Simmons et al., 2014; Pierson et al., 2018).

T lymphocytes (T-cells) are part of the adaptive immune system. Studies have shown that mechanical allodynia after nerve injury is associated with infiltration of the DRG by T-cells (Vicuña et al., 2015). However, preclinical studies in mice demonstrated that the active involvement of T-cells in the pathogenesis of neuropathic pain depends on the subtype of T-cells and the mouses gender (Sorge et al., 2015). Interestingly, T-cells also actively participate in the pain resolution process. After a period of hypernociception, the PNS and CNS can enter into a state of apparent deactivation (remission), also called “latent sensitization” (Marvizon et al., 2015). In this state, any noxious or stressful event leading to the reactivation of nociceptors, including the use of naloxone, will trigger a prolonged hyperalgesic response with changes in the expression of pain-related genes (Parada et al., 2003; Rivat et al., 2007; Price and Ray, 2019). T-cells and macrophages generate endogenous opioid peptides such as *β*-endorphins and enkephalins, which play a key role in preventing the reappearance of pain during latent sensitization (Stein et al., 1990; Ritter-Jones et al., 2016). Likewise, the anti-inflammatory cytokine IL-4, produced by mast cells, granulocytes, and T helper 2 (Th2) cells, exerts a modulatory effect on proinflammatory mediators and induces M2-like macrophages to release opioid peptides that attenuate pain (Celik et al., 2020).

Mast cells are located very close to nociceptive neurons; once activated, they release neuroactive proinflammatory cytokines (TNF-α, IL-1β, IL-6), chemokines, histamine, bradykinin, proteases, nerve growth factor (NGF), and substance P (SP). This release of proinflammatory cytokines generates the initial nociceptive signaling that modulates thermal and mechanical sensitivity through specific receptors (Chatterjee and Martinov, 2015). Crosstalk between mast cells and nociceptive neurons is established through their common receptor NK1. In addition, receptors located in the mast cells (S1P1, S1P2, and CRHR) interact with cytokines, chemokines, and NGF released by nociceptive neurons, sustaining the hyperactivation of nociceptors in the DRG and spinal dorsal horn and causing neurogenic inflammation (Kempuraj et al., 2019). Several authors have reported that substance P released from primary afferent nerve terminals not only interacts with its canonical receptor NK1 but can also activate the mast cell-specific receptor Mrgprb2 to promote the release of proinflammatory cytokines and chemokines, as well as facilitate the migration of immune cells to nociceptors (McNeil et al., 2015; Green et al., 2019). Additionally, NGF released by mast cells plays a primary role in peripheral sensitization by allowing the phosphorylation of transient receptor potential vanilloid 1 (TRPV1), which is a determinant factor in pain transduction. At the same time, TNF-α, IL-1β, and IL-6 also activate TRPV1 and other channel receptors like transient receptor potential ankyrin 1 (TRPA1), and sodium channels Nav1.7, Nav1.8, Nav1.9 in the nociceptor. Activation of these targets further contributes to peripheral sensitization and enhances signaling between nociceptor cell bodies in the DRG and the cerebral cortex where it will be processed as a pain sensation, subsequently resulting in central sensitization (Basbaum et al., 2009; Ji, et al., 2016; Pinho-Ribeiro et al., 2017; Locke et al., 2020).

Another determinant factor in the central sensitization process is the activation of Toll-Like Receptors (TLRs) in neurons and glial cells (Lacagnina et al., 2018; Zhang et al., 2020; Liu et al., 2022). Increased TLR signaling in macrophages, glial cells, and sensory neurons, accompanied by decreased action potential thresholds in nociceptive neurons, leads to increased nociceptive excitability and firing. The resulting exaggerated nociceptive response is a critical factor in the transition to persistent pain (Pinho-Ribeiro et al., 2017; Lacagnina et al., 2018; Locke et al., 2020). Furthermore, disruption of the BBB during the neuroinflammatory response allows for massive entry of immune cells into the CNS. The subsequent direct activation of nociceptive neurons and glial cells along the cerebral pathways in the brain worsens neuroinflammation and central sensitization, which perpetuates pain signaling, and facilitates the transition to chronic pain (Patel et al., 2015; Kempuraj et al., 2017; Gupta and IkkaHarvima, 2018; Mastrangelo et al., 2018).

In a rodent model, a proinflammatory phenotype similar to M1 in activated macrophages in the DRG was demonstrated, promoting the production of CGRP, which plays an active role in the persistence of the pain state (Massier et al., 2015).

### 2.2 Glial cell activation is essential in the transition from acute to chronic pain

Based on emerging evidence, the activation of glial cells is a phenomenon closely related to the origin and persistence of neuropathic and chronic pain, therefore it might be considered a “gliopathy” (Ji, Berta, and Nedergaard, 2013). Under normal conditions, the glial cells, especially microglia, provide microenvironmental conditions that promote neuronal development, synaptic pruning, and circuit formation, as well as the modulation of synaptic connectivity, neurotransmission, and neuroplasticity (Um, 2017). Microglial cells in the CNS emulate the phagocytic function of peripheral macrophages, helping in clearing the neural environment of damaged cells, microbial agents, and debris (Hanisch and Kettenmann, 2007). Astrocytes provide structural and metabolic support to glutamatergic synaptic transmission, regulating the extracellular concentration of glutamate (Vandenberg and Ryan, 2013; Ji et al., 2019). At the same time, oligodendrocytes speed up the synaptic transmission of the electrical impulse and are actively involved in pain in various ways. The production of IL-33 by oligodendrocytes in the spinal cord mediates the activation of microglial ST2 receptors in a mouse model of neuropathic pain (Malta et al., 2019). Glial cells also release anti-inflammatory cytokines that play an active role in repairing neurotoxic damage caused by neuroinflammation (Tiwari et al., 2014). Both in the DRG and in the trigeminal ganglion, satellite glial cells (SGCs) are located near the nuclei of nociceptive neurons, creating a neural structure unique to the PNS. Under normal conditions, this neuron-SGC coupling helps to maintain neuronal homeostasis, particularly by protecting axonic insulation and the integrity of the neural soma. Recent preclinical studies showed that SGCs have a key role in the neural repair process (Xiao et al., 2015; Hanani and Spray, 2020; Gazerani, 2021). Activation of SGCs after nerve damage leads to changes in K+ channels and increased release of cytokines and ATP (Mujenda et al., 2007). Immediately following nerve injury, there is an upregulation of the ATP receptor subtype P2X4 in spinal microglia, which influences microglial signaling to promote mechanical allodynia (Tsuda et al., 2003).

This functional relationship between neurons and SGCs contributes to the development of neuronal hyperactivity, and both peripheral, as well as the chronification of pain (Mujenda et al., 2007). After a peripheral nerve lesion, the degeneration produced by TNF-α and IL-1β release from the Schwann cells (SCs) significantly contributes to the progression to neuropathic pain (Fang et al., 2023).

Shortly after nerve damage, signs of microgliosis appear in the ipsilateral dorsal horn of the spinal cord within 2–3 days. It reaches peak levels in 4–7 days, before progressively declining weeks to months after the nerve lesion (Kohno et al., 2018). Spinal microglia activation originates from proinflammatory cytokines, chemokines, extracellular proteases, purines, excitatory neurotransmitters, and neuropeptides released by macrophages, natural killer cells (NK), and T-cells (Grace et al., 2014). T Helper 1 cells (Th1) also produce interferons (IFNs) that react with type 1FN receptors (IFNR) expressed by microglia, astrocytes, and neurons (Tan et al., 2021). IFNs vary in their effect when interacting with IFNR. IFNγ exhibits proinflammatory actions that activate glial cells and nociceptive neurons, contributing to the development of pain. IFNα and IFNβ promote the inactivation of microglia and astrocytes, as well as the inhibition of synaptic transmission in the spinal cord. In effect, they encourage restoration of the peripheral and central sensitization processes, leading to the resolution of neuropathic or chronic pain (Tsuda et al., 2009; Tan et al., 2021).

The complex array of inflammatory glial and immune mediators also includes other signaling molecules originating from the damaged nerve tissue, such as DAMPs and PAMPs (including LPS) (Ji et al., 2019; Gong et al., 2020; Jurga et al., 2020). Neural damage results in the overexpression of peripheral and central neurotrophic factors generated by neural damage, such as NGF, brain-derived neurotrophic factor (BDNF), and glial cell line-derived neurotrophic factor (GDNF) (Salio et al., 2014), neurotransmitters and neuropeptides (substance P, glutamate, CGRP) (Gwak et al., 2017). Additionally, activated astrocytes located in the presynaptic sensory neurons of the DRG and dorsal horns generate chemokines that intervene in microglial and oligodendrocyte activation (Chen et al., 2014; Ji et al., 2018; Ji et al., 2019; Liu et al., 2019; Malta et al., 2019). Upregulation of ATP receptor subtype P2X2 in spinal microglia immediately after nerve injury is also involved in microglial signaling that leads to mechanical allodynia (Tsuda et al., 2003). As mentioned before, once the microglial cells are activated, a highly dynamic process begins that leads to the production of multivariate morphological, metabolic, and functional states (Paolicelli et al., 2022) with a unique role in maintaining or repairing the neuroinflammatory state. These include the classically activated proinflammatory M1-like phenotype, and the intermittently activated M2-like phenotype microglia with anti-inflammatory and reparative effects (Willemen et al., 2014; Chen et al., 2018). M2-like phenotype microglia produce inflammation, cytotoxicity, and vascular changes leading to increased BBB permeability, and prolongation of the immune response (Jurga et al., 2020).

The synthesis and release of all these inflammation by-products in the PNS and CNS significantly affect synaptic transmission and interneuronal networking excitability, influencing the initiation and maintenance of pain (Coull et al., 2005; Ji et al., 2006; Pezet and McMahon, 2006; Kawasaki et al., 2008; Vezzani and Viviani, 2015). The resulting neuroinflammatory state creates an exaggerated afferent input that promotes changes in plasticity in nociceptive neurons and synaptic transmission at the spinal cord level, increasing hyperexcitability of nociceptors, enhancement of signaling transduction to the brain and facilitating the development of central sensitization, which perpetuates the pain state and the transition to neuropathic or chronic pain (von Banchet et al., 2009).

### 2.3 The resolution phase of the neuroinflammatory damage

After peripheral nerve injury, transected axons elicit degeneration of the distal nerve endings and in the axotomized nociceptors bodies in the DRG. The post-injury neuroinflammation state that occurs can originate deleterious consequences (neuropathic and/or chronic pain) and beneficial effects triggering reparative processes in the injured peripheral nerve as well as in the DRGs nociceptive neurons. The axonal regeneration and neuron functional recovery is not an autonomous process and depends on immune cells, especially macrophages, and glial cells (Schwann cells). On day 3 after nerve injury, macrophages accumulate, most at the distal end and less proximal to the lesion (Taskinen and Roytta, 1997). Almost at the same time (day 4), macrophage accumulation can be detected at the DRG (Lu and Richardson, 1993). This noxious event activates chemokines CCL2 in the nerve injury region, Schwann cells, and in the DRG, which binds to CCR2, G-protein-coupled receptors located in the macrophages, attracting them to the distal nerve cell body area as well as to the DRG (Ransohoff, 2009). Macrophages and Schwann cells facilitate the removal of axon debris, myelin phagocytosis, and the clearance of molecules from degenerating axons that inhibit neural regeneration and axonal outgrowth in the nerve endings and the DRG (Zigmond and Echevarria, 2019). Post-traumatic neural regeneration is almost an exclusive property of the PNS, however, not all injured axons achieve complete regeneration (Gordon et al., 2009) since the neural repair mechanism in the PNS is not only slow but often incomplete. Resident macrophages are cells found in peripheral nerves and ganglia while infiltrating macrophages access the neural tissue after a nerve injury or infection, outnumbering the resident macrophages. Although resident macrophages are the “first responder cells” after nerve injury, later, infiltrating macrophages outnumber them (Mueller et al., 2003). Unlike peripheral axonal damage or transection, there is no macrophage accumulation with crushing damage to the dorsal root neurons, in which regeneration of centrally projecting axons does not occur (Kwon et al., 2013).

During the resolution phase that follows the initial post-injury activation, M1-like macrophages can transition into M2-like macrophages (Van der Bossche et al., 2016). Several recent studies showed that infiltrating macrophages attracted to the nerve injury and to the DRG express M2-like subtype which not only release antiinflammatory cytokines (IL-4, IL-10, IL-13, and TGF-β) depending upon the pathologic state (Willemen et al., 2014). M2-like macrophages also release other mediators that promote repair of the axonal and neuron damage such as neurotrophic factors, growth factors, colony-stimulating 1 (CSF-1), and progranulin (Kwon et al., 2013; Martinez and Gordon, 2014; Wynn and Vannella, 2016; Jurga et al., 2020). Oncomodulin, secreted by macrophages and granulocytes, promotes neuron outgrowth in axotomized sensory neurons in the DRG (DeFrancesco-Lisowitz et al., 2015; Kwon et al., 2013). *In vitro* studies demonstrated that cytokine IL-1β secreted by macrophages stimulates the production of NGF and other neurotrophins such as BNDF, NT3, and NT4/5, which enhances regeneration at the distal nerve stump (Barrette et al., 2008). A recent study conducted by Feng et al. reported that the self-renovation of resident macrophages in the DRG is a contributing factor to axonal regeneration very similar to the self-renewal of glial cells in the brain (Feng et al., 2023).

Microglial polarization into the M2-like phenotype is also stimulated by the presence of IL-4 and IL-13 cytokines produced by T-cells (Th2) (). After activation, the M2-like microglial phenotype releases antiinflammatory cytokines (IL-10 and TGFβ), (Saijo et al., 2013; Jurga et al., 2020).

M2-like phenotype microglia plays a decisive role in repairing neural damage by inhibiting neuroinflammation, restoring neuronal homeostasis, removing cellular debris through phagocytosis, and protecting the extracellular matrix (Jurga et al., 2020).

Other components involved in the resolution phase of neuroinflammation are resolvins and protectins, which are a group of molecules derived from omega-3 fatty acids. Resolvins and protectins represent one part of a biochemical arsenal that functions to restore homeostasis once the initial inflammatory response is over (Sommer and Frank, 2011). New data suggest these substances might have future applications as analgesic drugs that reduce inflammatory pain by blocking TRP channels and NMDA receptors in somatosensory neurons located in the dorsal horn of the spinal cord (Ji et al., 2011; Sommer and Frank, 2011). Additionally, resolvins and protectines exhibit a neuromodulator-like profile that affects only pathological pain sensations, but not normal sensations evoked by painful stimuli (Ji et al., 2016; Roh et al., 2020).

The transition from acute to chronic pain can start shortly after the event that triggered the onset of the acute pain (2–3 weeks) (Price and Ray, 2019). Effective modulation of the neuroinflammation and initiation of the restorative process requires a balanced expression of M1-like and M2-like microglial phenotypes. A recent preclinical study by Li et al. in rats revealed that activated microglia mediate the transformation of spinal cord astrocytes predominantly into the A1 phenotype, which promotes neuroinflammation and neurotoxicity, and favors the appearance of CPSP, and to a lesser extent into the A2 phenotype, which provides neuroprotection and restoration (Li et al., 2020). The existing preclinical and clinical evidence suggests that after this transitional process, the maintenance of the chronic pain state is mainly attributed to central sensitization; however, recent clinical studies using neuroimaging have shown that peripheral nerve blocks in patients with neuropathic pain provide pain relief. This suggests that sustained primary afferent output from nociceptors and altered neuroplasticity, characterized by hyperexcitability and over-signaling in the synaptic relays at the spinal cord play a crucial role in the development of chronic and neuropathic pain after surgery (Haroutiunian et al., 2013; Haroutiunian et al., 2014; Vaso et al., 2014; Ratte and Prescott, 2016).

## 3 Importance of the enteric nervous system in acute and chronic pain

**FIGURE 2:**
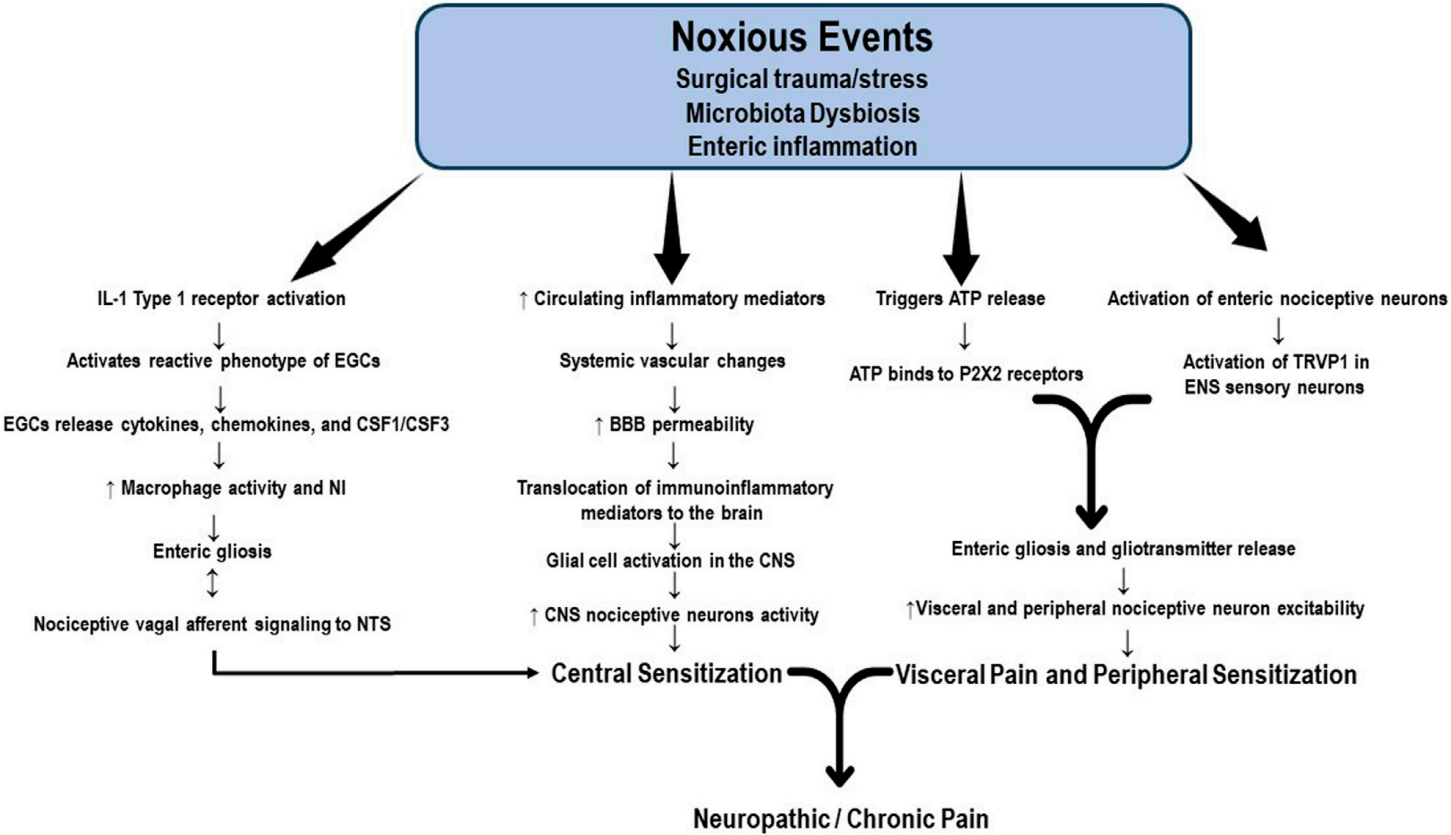
Potential mechanisms of enteric gliosis. Noxious events, including surgical trauma and bowel surgery, produce enteric dysbiosis and activation of the IL-1 receptor, which induces a reactive phenotype of enteric glial cells (EGCs), a process called enteric gliosis. EGCs release cytokines, chemokines, and colony-stimulating factors 1 and 3 (CSF1, CSF3), key elements modulating macrophage activation. The crosstalk between EGCs and macrophages is crucial to enteric neuroinflammation and visceral afferent nerve hyperactivity. Vagal afferent neurons (VAN) are the primary enteric nociceptive neurons that integrate gut signaling and connect VAN via the vagus nerve to the NST in the brainstem. Enteric dysbiosis modulates inflammation in the afferent vagal nerve and the transmission of noxious information from the gut to the brain, contributing to central sensitization. The visceral nociceptive neurons and VAN hyperactivity also induce nociceptor sensitivity in the peripheral nervous system. Surgical trauma triggers the release of ATP, which ultimately binds to purinergic receptors (P2XR). This signaling pathway represents another factor responsible for the visceral hypersensitivity and chronic abdominal pain associated with enteric gliosis. Gliotransmitter release activates the mesenteric plexus enteric nociceptive neurons and TRVP1 sensory neurons. Nociceptor activation and the release of immunoinflammatory mediators may persistently elevate for weeks or months, leading to the transition to chronic or neuropathic pain. **BBB:** brain blood barrier; **CSF1:** colony-stimulating factor 1; **CSF3:** colony-stimulating factor 3; **EGCs:** enteric glial cells; **NST:** nucleus solitary tract; **P2X2:** purinergic 2 × 2 receptor; TRVP1: transient receptor potential vanilloid receptor.

Increasing evidence supports the role of continuous functional interdependence of the microbiota, enteric nervous system (ENS), PNS, and CNS. The so-called microbiota-gut-brain axis is composed of the colonic myenteric plexuses, the dorsal root ganglion, the nucleus solitary tract (NST), and the periaqueductal grey. It constitutes an important factor in the pathogenesis of acute visceral, nociplastic, neuropathic, and chronic pain; however, most studies remain in the realm of research in animal models of pain (Harotounian et al., 2014; Vaso et al., 2014; Ratte and Prescott, 2016). The intrinsic structure of the ENS consists of a neural network of resident neurons and glial cells located in the intestinal mucosa and the inner muscularis propria. The ENS integrates and transduces immune, inflammatory, and neuroendocrine signals that reach the brain tissue through the vagus nerve and the BBB, producing alterations in its permeability and allowing the translocation of immune and inflammatory mediators to the brain tissue. Current evidence also suggests that enteric neuronal plasticity and glial activation are fundamental players in the development of neuropathic and chronic pain (Morales-Soto and Gulbransen, 2019). The alleged mechanisms proposed to activate enteric neurons and glial cells include the production of immune and inflammatory mediators and neuromodulatory by-products of bacterial metabolism (Grubišić and Gulbransen, 2017). Enteric neuronal plasticity is directly involved in sensitizing visceral afferent sensory nerve fibers, augmenting neural sensitivity, and increasing firing patterns in the peripheral pain neural networking, spinal cord, and brain, which are all regulated by glial cell hyperactivity (Grubišić and Gulbransen, 2017; Morales-Soto and Gulbransen, 2019). Subtle differences in microbiota composition, amino acid levels, and neurotransmitters have been detected in individuals suffering from several chronic pain syndromes, such as fibromyalgia (Clos-Garcia et al., 2019; Ji et al., 2019; Minerbi et al., 2019; Bistoletti et al., 2020). Qualitative and quantitative alterations in the microbiota, or dysbiosis, stimulate the production and release of proinflammatory substances by enteric immune and glial cells. Some noxious events, including surgical trauma and bowel surgery, produce dysbiosis and activation of IL-1 receptor type I (IL-1R1), which induces a reactive phenotype of enteric glial cells (EGCs) called enteric gliosis (Schneider et al., 2021). Once activated, EGCs release cytokines, chemokines, and colony-stimulating factors 1 and 3 (CSF1, CSF3) which are key elements modulating macrophage activation (Grubišić and Gulbransen, 2017; Schneider et al., 2022). The bidirectional communication between EGCs and macrophages is crucial to enteric neuroinflammation and visceral afferent nerve hypersensitivity (Schneider et al., 2022). Vagal afferent neurons (VAN) are the enteric primary nociceptive neurons that integrate gut signaling and connect VAN via the vagus nerve to the NST in the brainstem. In this way, dysbiosis modulates inflammation in the afferent vagal nerve and the transmission of noxious information from the gut to the brain (Kim et al., 2020). The visceral nociceptor neurons and VAN hyperactivity also induce nociceptor sensitivity in the PNS, which is substantive in generating chronic visceral pain and transitioning from acute to chronic pain. Schneider et al. recently identified another potential pathway for enteric gliosis and neuroinflammation following intestinal surgery. According to them, surgical trauma triggers ATP release that binds to purinergic receptors (P2X receptors), drives enteric gliosis and intestinal inflammation, and is mainly responsible for visceral hypersensitivity and abdominal pain (Schneider et al., 2021) Although not wholly studied, enteric gliosis and gliotransmitter release have been suggested to be closely associated with the activation of enteric nociceptive neurons and TRVP1 sensory neurons in the mesenteric plexus, contributing to the development of prolonged visceral pain (Figure 2). However, the mechanisms involved in the changes in sensitivity in the PNS and CNS resulting from the interaction of gliotransmitters with enteric nociceptors remain largely unknown (Xu et al., 2008; Morales-Soto and Gulbransen, 2019). After the neuroinflammatory response accompanying the initial neural injury, the healing process begins and promotes tissue restoration, neutrophil apoptosis, and scarification. However, nociceptor activation and the release of immunoinflammatory mediators may persist elevated for weeks or months, leading to the transition to chronic or neuropathic pain (Chavan et al., 2017).

## 4 Neuroinflammation associated with opioids

The belief that opioids exert their effects solely by binding to their receptors does not adequately support the pharmacological basis of their use as pain control agents in clinical practice. The appearance of undesirable effects that compromise analgesic efficacy, such as hyperalgesia, allodynia, tolerance, and increased opportunistic infections, particularly in prolonged use scenarios, suggests the involvement of other mechanisms. This section considers some aspects of the immune response as elements of the opioid-analgesic equation. It is important to note that interactions between the nervous and immune systems are not exclusively limited to inflammatory conditions. Communication between these systems also occurs as a consequence of signaling crosstalk between immunocompetent cells, glia, endothelial cells, and neuronal cells in peripheral and central locations, and ultimately alters neuronal function (Watkins et al., 2007; Shah and Choi, 2017; Morioka et al., 2019). It is also important to recognize that pain processing is not simply the result of signals traveling from a damaged zone to the brain cortex but the confluence of multiple dynamic influences that can enhance or suppress nociceptive messages (Milligan and Watkins, 2009). Before unraveling the complex relationship between opioids and neuroinflammation, the beneficial analgesic actions of opioids must be separated from the detrimental interactions that underlie their undesirable effects. Additionally, it must be remembered that drugs currently used to treat clinical pain conditions were developed to target neurons before the discovery of these complex interactions that compromise opioid efficacy (Watkins et al., 2007).

The seminal work of Goldstein et al. brought to the forefront that opioids could also nonselective bind to non-opioid receptors (Goldstein et al., 1971). Years after, Wybran et al. reported that morphine possessed an immunosuppressive effect that could be modulated by naloxone, a classic antagonist of opioid activity (Wybran et al., 1979). Later, Watkins et al. published an elegant paper reporting that morphine administration caused glial activation in the spinal cord. As a result, it led to the release of substances that antagonize opioid action while causing pro-inflammatory effects and inducing a central immune response (Watkins et al., 2005). The participation of non-neuronal cells in these mechanisms further complicates the pharmacology of opioids.

**FIGURE 3:**
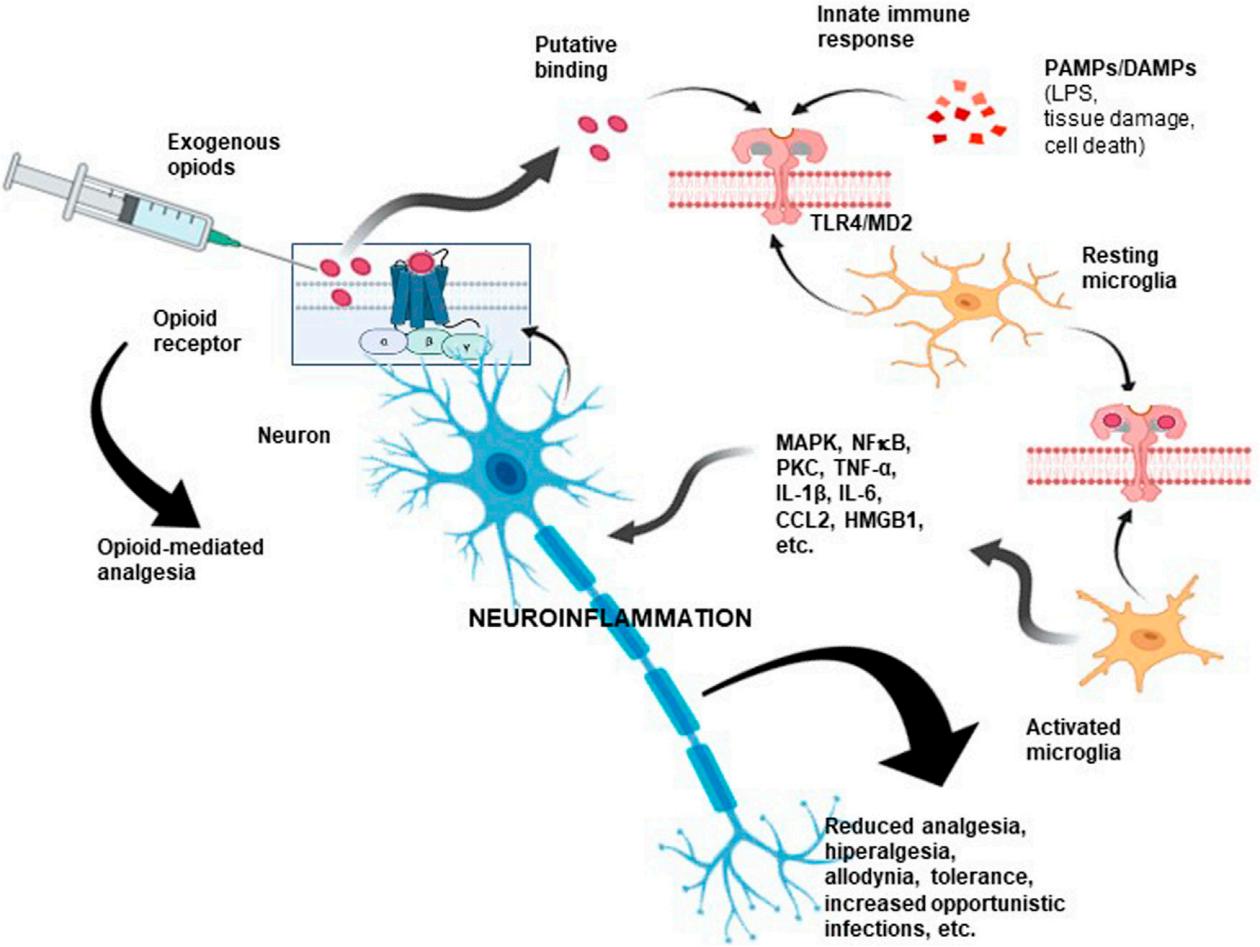
Opioid-associated neuroinflammation. As a consequence of opioid administration, proinflammatory scenarios become activated via TLR4/MD-2. As shown, opioids could also bind nonselectively to a non-opioid receptor due to interactions with critical sites shared by LPS, some PAMPs, and DAMPs, inducing downstream signaling. These interactions may compromise analgesic efficacy and cause unwanted opioid side effects. Hence, an opioid could be considered a proinflammatory molecule. This type of neuroimmune interaction could partly explain the unwanted side effects observed after prolonged administration of opioids, including the development of tolerance, allodynia, paradoxical hyperalgesic effects, and even the induction of a proinflammatory response. **TLR4**: toll-like receptor 4; **MD-2**: myeloid differentiation factor 2; **PAMPs**: pathogen-associated molecular pattern molecules; **DAMPs**: damage-associated molecular pattern molecules.

Research aimed at clarifying this less-known opioid action identified TLR4 as a critical element of this “atypical” signaling (Figure 3). This transmembrane receptor belongs to the Toll-like receptor (TLR) family, which activates the innate immune response by recognizing PAMPs (from viruses, bacteria, protozoa, and fungi) or DAMPs as a consequence of tissue damage or cell death (Moresco et al., 2011; Zhang et al., 2020). In an interesting study, Grace et al. suggested that morphine administration leads to the persistent release of DAMPs with actions mediated by TLR4 and the purinergic receptor P2X7R (Grace et al., 2016).

LPS, a component of the cell wall of Gram-negative bacteria, is a classic exogenous TLR4 agonist. However, a comprehensive review by Zhang et al. showed evidence that opioid receptor agonists directly activate TLR4, even in the absence of LPS (Zhang et al., 2020). Of note, this property is also shared with endogenous opioids, which, by the way, can be derived from immune cells. Unlike the interaction between opioid and opioid receptors, the binding of opioids to TLR4 is not stereoselective. Interestingly, morphine-3-glucuronic acid (M3G), an inactive metabolite without affinity for opioid receptors, also activates TLR4 (Wu et al., 2007; Hutchinson et al., 2010).

The high mobility group box 1 protein (HMGB1), an endogenous TLR4 agonist, is an alarmin that acts in synergy with endogenous and exogenous danger signals to promote inflammation. HMGB1 is secreted not only by reactive immunocompetent cells but also by neurons and glial cells (Takeuchi and Akira, 2010; Shah and Choi, 2017). It is important to note that TLR4 has been associated with morphine-induced HMGB1 production (Qian et al., 2020) and that HMGB1 is associated with abnormal pain processing and development of tolerance, hyperalgesia, and allodynia (Zhang et al., 2020).

Myeloid differentiation factor 2 (MD2), is an essential component of the TLR4 signaling receptor complex that recognizes and initiates an innate immune response to bacterial LPS. Opioid binding to MD2 activates signaling processes that lead to increased expression of the nuclear factor kappa-light-chain enhancer of activated B cells (NF-kB) and the production of pro-inflammatory cytokines such as TNF-a, IL-1β, and IL-6 (Takeuchi and Akira, 2010; Liu et al., 2022). Furthermore, opioid and TLR4 signaling activates the mitogen-activated protein kinase (MAPK) pathway, leading to neuroinflammation (Zhang et al., 2020). These pieces of evidence suggest that opioids may function as proinflammatory-like cytokines causing neuroinflammatory and immunosuppressive effects. It also highlights potential intracellular crosstalk mechanisms between immune cells, glial products, and neurons (Wang et al., 2012; Grace et al., 2014; Grace et al., 2015).

As suggested above, the interaction between opioids and the TLR4 receptor may also explain the hyperalgesia, allodynia, and tolerance observed after morphine administration, which has been associated with opioid-induced proinflammatory glial activation (Hutchinson et al., 2010). Evidence indicates that opioid-induced immune effects could explain the drugs decreased efficacy, including the critical role that non-neuronal immunocompetent cells, such as astrocytes and microglia, could play (Shah and Choi, 2017). As discussed earlier, the endogenous TLR4 agonist HMGB1 can translocate from the nucleus to the cytoplasm or the extracellular space. The binding of transmembrane TLR4 initiates the innate immune response, which activates NF-κB and mediates the transcription of pro-inflammatory cytokines in macrophages, monocytes, and glial cells. HMGB1 binds to several receptor systems, including TLR2, TLR4-5, and the receptor for advanced glycosylation end products (RAGE). All these receptors are implicated in the antianalgesic effects of opioids (He et al., 2013; Das et al., 2016; Morioka et al., 2019). Prior work has demonstrated that morphine administration increases HMGB1 expression and release. Additionally, it has been shown to promote mechanical allodynia, which may be sustained for months after the discontinuation of morphine treatment. In this setting, investigators noted an increase in IL-1β release from activated spinal microglia and the NOD-like receptor protein 3 (NLRP3) inflammasome (Grace et al., 2016), a cytosolic multiprotein oligomer of the innate immune system responsible for activating inflammatory responses. Interestingly, this multiprotein complex activates IL-1β through proteolytic cleavage by caspase-1. TLR4 has also been associated with opioid-induced hyperalgesia via protein kinase C (Araldi et al., 2019). It should be noted that the involvement of TLR4 in the antianalgesic effects of opioids has also been demonstrated in TLR4 mutant and knockout mice (Mattioli et al., 2014), suggesting that perhaps anti-opioid activity does not depend exclusively on TLR4 but can be complemented and facilitated by this receptor.

As we already know, pain induced by a stimulus or pathological damage sets in motion multiple interactions in the neuronal environment. The appropriate knowledge of these multipartite interactions might help avoid vicious cycles that cause pain persistence and counteract the analgesic effect of opioids. Such interactions may also be triggered by opioid administration alone, disrupting the innate response to infections and tissue damage. Therefore, we must expand our knowledge of analgesic pharmacology and the related neuroimmune responses. This might help us to understand that the apparent pronociceptive incongruities observed after sustained opioid administration have a reason to exist. These discrepancies become an interesting paradox when selecting the most appropriate analgesic for pain management. Fortunately, the contributions of recognized research teams are opening new avenues for deciphering these entangled concepts. They are also expanding frontiers in pain management. Specifically, they beckon the future development of therapeutic agents that target immune-neuronal-glial interactions during pain processing without inhibiting these interactions restorative and protective properties. Additionally, they reveal opportunities to design new analgesic molecules that may avoid non-neuronal sites while retaining their original neuronal binding properties. Perhaps the solution to these problems lies in maintaining an adequate homeostatic balance among the cellular constituents that inhabit the same neighborhood in which neurons exist. Most undesirable effects of opioids are not evident under basal conditions in glial and immunocompetent cells.

## 5 Summary and conclusion

This narrative review was undertaken to address the complex cellular mechanisms involved in the genesis of neuroinflammation as a fundamental factor contributing to the progression and chronicity of pain as well as the immunoinflammatory response resulting from the activation of glial cells by opioids, which aggravates the neuroinflammatory process and causes the development of tolerance, dependence, or paradoxical hyperalgesia that interferes with the analgesic effects of opioid drugs. We also sought to identify the most recent evidence supporting the usefulness and effectiveness of alternative non-pharmacological interventions, such as regulation of the microbiota-gut-brain axis, which can also modulate glial cell activation and create more effective multimodal synergistic therapies for patients with chronic pain syndromes, improving their clinical and functional outcomes.
